# Removal of Steatomatous Tumor

**Published:** 1876-11

**Authors:** 


### ARTICLE IX.

### *Removal of Steatomatous Tumor.*

This operation was performed on the the 2d of August, 1876. The patient was Mr. B. of———, Indiana, a young man 24 years of age. Dr. H. E. Munn, formerly of Waterbury, Conn., assisted and reported the operation.

The tumor was situated in the right anterior superior cervical triangle, extending from the mastoid process of the temporal bone nearly to the thyroid cartilage. In shape, it was somewhat reniform with the inferior extremity dipping in beside the cartilage.

This was originally an encysted tumor of the variety described by the authorities as atheromatous or steatomatous, probably occasioned by the occlusion of one of the sebaceous folliciles and containing sebaceous matters; but by officious and ignorant interference, during a period of four years, in the way of puncturing and the application of irritating substances, inflammatory action had been from time to time excited and the capsule had assumed as enchondromatous character, while the contained matters had degenerated into a sanious pus.

Adhesions had formed with the mastoid process, the integument by several cicatrices, a part of the sterno-cleido-mastoid muscle and the parotid gland. It was about the size of a goose egg, and was separated only by areolar tissue from the internal and external jugular vein, their communicating branch and the posterior auricular vein and the external carotid artery.

The operation was performed by making two incisions, forming an ellipse three inches long and three-fourths of an inch wide, commencing half an inch below the ear and terminating a little below the angle of the lower jaw, enclosing one of the cicatrices and a fistulous canal leading to the centre of the tumor, produced by previous treatment. Incisions were then made laterally, and flaps of integument and superficial fascia were dissected up, exposing the tumor, which was then elevated with the tenaculum. Owing to the firmness of the adhesions and the irregular shape, careful dissections were necessary to complete the operation.

The hemorrhage during the operation was quite extensive—although the time occupied did not exceed three minutes—owing to the multiplicity and engorgement of the arterioles and venous radicles supplying the growth.

The capsule upon its secreting surface presented a granular or lobulated appearance and was somewhat cartilaginous in texture. It was invested with a dense tissue containing fibrous and areolar layers interspersed with fat.—*New York Eclectic Medical News.*

